# Case report: A rare case of synchronous mucinous neoplasms of the renal pelvis and the appendix

**DOI:** 10.3389/fonc.2023.1213631

**Published:** 2023-06-26

**Authors:** Yuhua Zou, Xiaojuan Xie, Qinlin Wang, Cunzhi Zhong, Quanliang Liu

**Affiliations:** ^1^ Department of Urology, The First Affiliated Hospital of Gannan Medical University, Ganzhou, China; ^2^ Department of Cardiology, The First Affiliated Hospital of Gannan Medical University, Ganzhou, China; ^3^ Department of Anesthesiology, Operation Rom, The First Affiliated Hospital of Gannan Medical University, Ganzhou, China

**Keywords:** mucinous neoplasm, appendiceal mucinous neoplasm, kidney, renal pelvis, pyonephrosis, laparoscopic nephrectomy, case report

## Abstract

**Background:**

Mucinous neoplasms are tumors arising in the epithelial tissue, characterized by excessive mucin secretion. They mainly emerge in the digestive system and rarely in the urinary system. They also seldom develop in the renal pelvis and the appendix asynchronously or simultaneously. The concurrence of this disease in these two regions has not yet been reported. In this case report, we discuss the diagnosis and treatment of synchronous mucinous neoplasms of the right renal pelvis and the appendix. The mucinous neoplasm of the renal pelvis was preoperatively misdiagnosed as pyonephrosis caused by renal stones, and the patient underwent laparoscopic nephrectomy. Herein, we summarize our experience with this rare case in combination with related literature.

**Case presentation:**

In this case, A 64‐year‐old female was admitted to our hospital with persistent pain in the right lower back for over a year. Computer tomography urography (CTU) showed that the patient was confirmed as right kidney stone with large hydronephrosis or pyonephrosis, and appendiceal mucinous neoplasm (AMN). Subsequently, the patient was transferred to the gastrointestinal surgery department. Simultaneously, electronic colonoscopy with biopsy suggested AMN. Open appendectomy plus abdominal exploration was performed after obtaining informed consent. Postoperative pathology indicated low-grade AMN (LAMN) and the incisal margin of the appendix was negative. The patient was re‐admitted to the urology department, and underwent laparoscopic right nephrectomy because she was misdiagnosed with calculi and pyonephrosis of the right kidney according to the indistinctive clinical symptoms, standard examination of the gelatinous material, and imaging findings. Postoperative pathology suggested a high‐grade mucinous neoplasm of the renal pelvis and mucin residing partly in the interstitium of the cyst walls. Good follow-up results were obtained for 14 months.

**Conclusion:**

Synchronous mucinous neoplasms of the renal pelvis and the appendix are indeed uncommon and have not yet been reported. Primary renal mucinous adenocarcinoma is very rare, metastasis from other organs should be first considered, especially in patients with long-term chronic inflammation, hydronephrosis, pyonephrosis, and renal stones, otherwise, misdiagnosis and treatment delay may occur. Hence, for patients with rare diseases, strict adherence to treatment principles and close follow‐up are necessary to achieve favorable outcomes.

## 1 Introduction

Mucinous neoplasms are tumors that mostly originate in the epithelial tissue and secrete mucin excessively. They commonly emerge in the gastrointestinal tract, as well as in the ovaries and breasts, and they rarely develop in the urinary system (1). Appendiceal mucinous neoplasm (AMN) is a rare type of neoplasm affecting the digestive system, accounting for merely 0.2%–0.3% of all appendectomy specimens and causing atypical clinical symptoms during its early onset (2). Tumors of the renal pelvis mostly originate in the transitional epithelium, and 90% of the cases are urothelial carcinoma. Mucinous neoplasms of the renal pelvis represent only 1% of all malignant cases, thereby rarely reported (3); consequently, making a preoperative diagnosis is rather challenging, and the risk of misdiagnosis is high because of the lack of typical clinical, laboratory, and imaging findings (4, 5). Seeing as a research gap, we report a distinctly rare case of synchronous mucinous neoplasms of the renal pelvis and the appendix. In this case, the mucinous neoplasm of the renal pelvis was preoperatively misdiagnosed as pyonephrosis induced by renal stones. This case report aims to broaden the understanding of the disease and develop standard diagnosis and treatment approaches.

## 2 Case presentation

**Figure 1:**
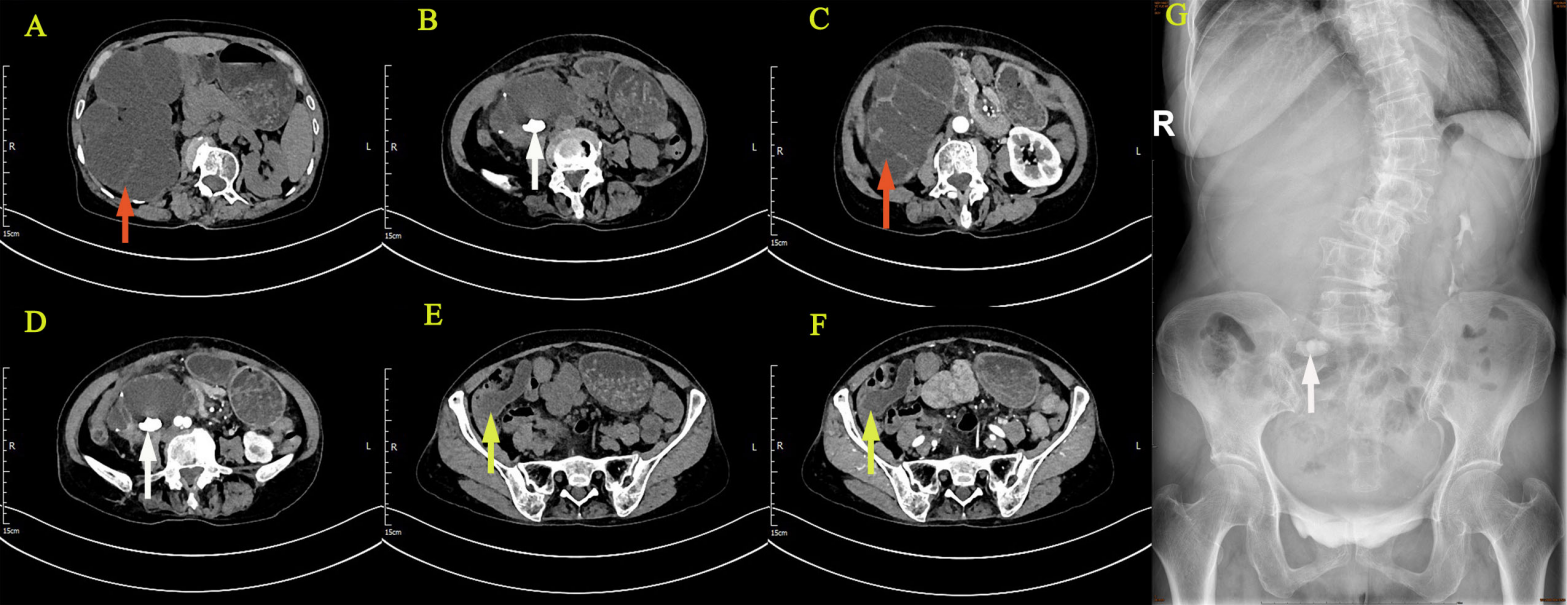
Imaging examination: CTU suggested right kidney enlargement **(A–D)**, multiple hyperdense nodules **(B, D)**, the contrast-enhanced urogram showed significant enlargement and dilatation of the right kidney and the upper segment of the right ureter **(C)**. The appendix also thickened with mild enhancement **(E, F)**, with a hypodense lumen without enhancement **(F)**. IVU showed no development of the right kidney, right kidney stone, and normal excretion of the left kidney **(G)**. (The giant right kidney was showed by red arrows, the kidney stone was showed by white arrows, and the appendix was showed by yellow arrows.).

**Figure 2:**
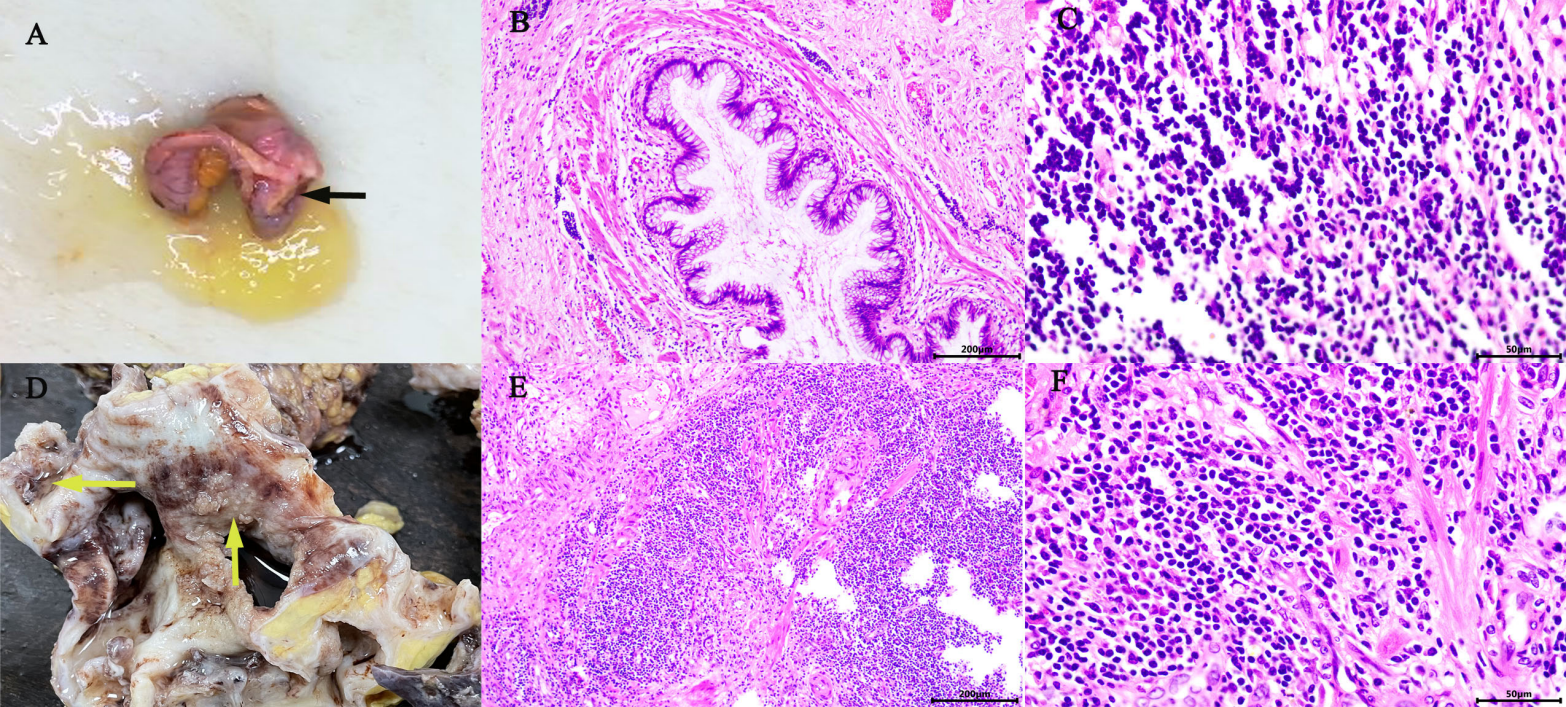
Gross appearance: The appendix was swollen and thickened, filled with mucus (black arrow) **(A)**. The right kidney was found: dilated renal pelvis, thin renal parenchyma, multilocular and dilated renal cysts with abundant gelatinous mucus and cauliflower-like masses in the renal pelvis and calyces (yellow arrow) **(D)**. Microscopic overview of the appendix **(B, C)** and the right kidney **(E, F)**. [Hematoxylin-eosin staining, ×100 **(B, E)**, ×400 **(C, F)**].

The reporting of this study conforms to CARE guidelines (6). A 64‐year‐old female with a body mass index (BMI) of 20.64 kg/m^2^ was admitted to the urology department on June 22, 2021, complaining of persistent pain in the right lower back for over a year. The patient described the pain as intermittent, dull, and sore, occasionally accompanied by chills, low‐grade fever (highest temperature: 37.8°C), abdominal distension, nausea, and vomiting. Abdominal pain, migratory pain in the right lower abdomen, urinary frequency, urgency, dysuria, gross hematuria, rectal bleeding, and purulent bloody stool were not reported. The patient also had no history of smoking, abdominal or lumbar surgery, or extracorporeal shockwave lithotripsy (ESWL). Physical examination revealed mild bulging in the right abdomen and tenderness on percussion over the right kidney. Preoperatively, urinalysis was negative for red blood cells and protein but positive for white blood cells (2+); hemanalysis and comprehensive metabolic panel showed no significant abnormalities; tumor marker tests revealed 25.7 ng/ml for carcinoembryonic antigen (CEA) and 33 U/ml for carbohydrate antigen 19-9 (CA‐199). Computed tomography urography (CTU) suggested right kidney enlargement, multiple hyperdense nodules measuring approximately 23 × 10 mm in the right renal calyces, and right ureteral wall thickening. In addition, the contrast-enhanced urogram showed significant enlargement and dilatation of the right kidney and the upper segment of the right ureter. The appendix also thickened with mild enhancement, measuring approximately 14 mm in diameter, with a hypodense lumen without enhancement. Working diagnoses were i) right ureteral wall thickening, suggestive of inflammatory changes, multiple stones in the right kidney, and severe hydronephrosis of the right kidney; and ii) abnormal appendiceal density, suggestive of a mucinous tumor (**Figures 1A–F**). Intravenous urography (IVU) showed no development of the right kidney, right kidney stones, normal excretion of the left kidney, and scoliosis (**Figure 1G**). Computed tomography (CT) of the chest showed no mass lesions. The estimated glomerular filtration rates (eGFR) were 62.08 and 15.48 mL/min for the left and right kidneys, respectively. On the same day, the patient underwent ultrasound-guided percutaneous right nephrostomy under local anesthesia; consequently, a large amount of gelatinous material was drained. Furthermore, the standard analysis showed that the gelatinous material was negative for red blood cells and bacterial culture but positive for white blood cells (2+). Cystoscopy showed purulent deposits in the bladder. Although the diagnosis of a neoplastic lesion in the appendix was confirmed, we could not establish a definite diagnosis of the right kidney with suspected pyonephrosis preoperatively. The patient’s family was informed of her condition and requested to prioritize the treatment for this neoplasm. Therefore, the patient was transferred to the gastrointestinal surgery department for further evaluation. Electronic colonoscopy with biopsy suggested AMN. Exfoliative cytology revealed numerous degenerated cells without apparent atypia in three consecutive tests, the results were negative, and on July 2, open appendectomy plus abdominal exploration was performed under general anesthesia. The appendix was completely removed, and upon specimen collection, a great amount of mucus was seen in the appendix cavity (**Figure 2A**). Postoperative pathology indicated low-grade AMN (LAMN) and the incisal margin of the appendix was negative (**Figures 2B, C**). As the patient decided to defer further treatment for the right kidney, she was discharged for rest and recovery.

**Figure 3:**
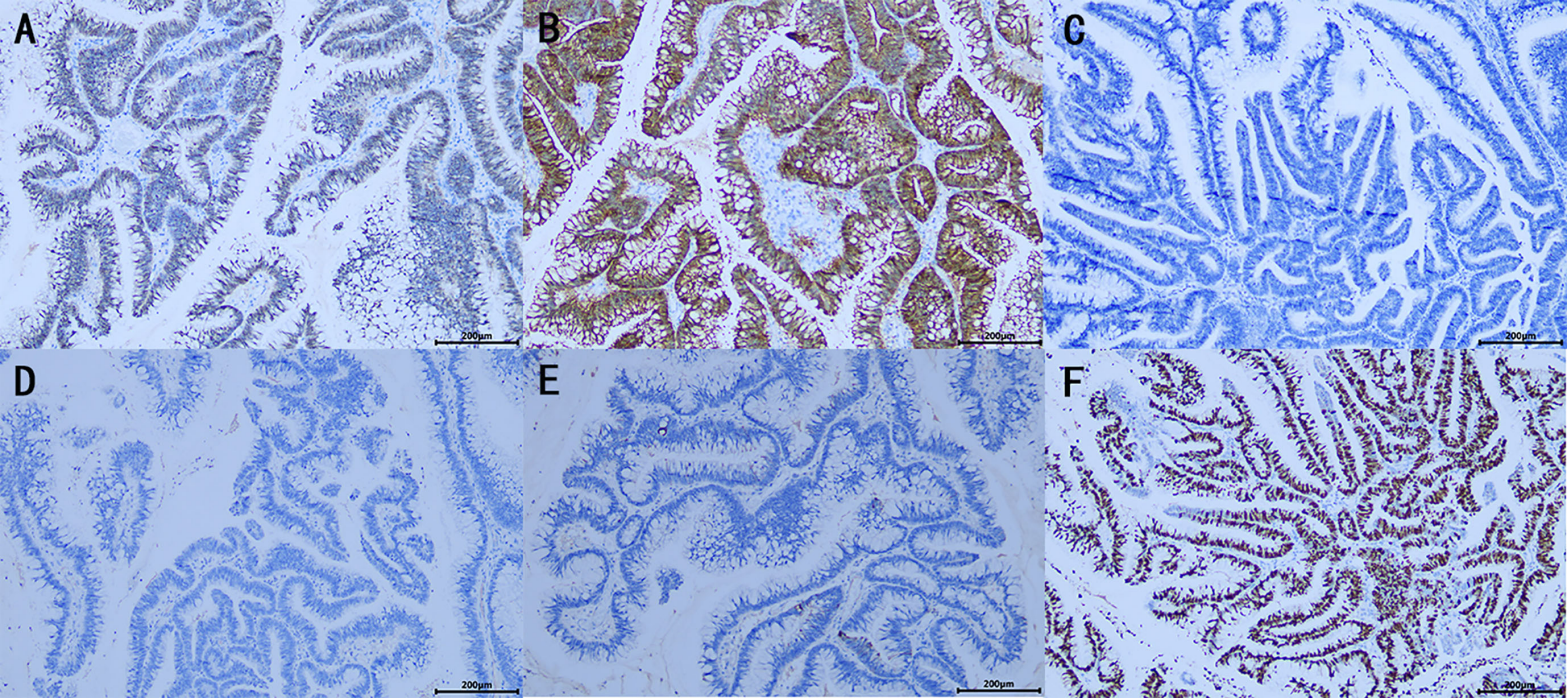
Immunohistochemistrical (IHC) analysis **(A–F)**: **(A)** CDX2 (+), **(B)** Villin (+), **(C)** GATA3(−), **(D)** P63 (−), **(E)** CK7 (−), **(F)** Ki-67 (about 60%+), indicated features of appendiceal mucinous neoplasm metastasis to the renal pelvis. (×100).

On October 13, 2021, the patient was re‐admitted to the urology department for right kidney treatment. Follow-up abdominal CT scans with contrast enhancement showed multiple stones, severe hydronephrosis, and thickening of both the renal pelvis and the ureteral wall of the right kidney. Infectious lesions were considered. The appendix was not shown. Other abdominal parts were unremarkable. At the same time, the patient and her family agreed to laparoscopic right nephrectomy under general anesthesia. The right kidney was severely attached to the lateral peritoneum and surrounding tissues, making it difficult to be removed. Although the surgery went smoothly without conversion to open surgery or causing any incidental injuries, the operation lasted for approximately 230 min, with a blood loss of 200 ml. Postoperatively, 0.4 g of 5‐fluorouracil and 500 ml of normal saline were infused once intraperitoneally for 2 h. The affected kidney and upper ureter remained intact after being removed from the body. When the kidney was sliced open, the following were found: dilated renal pelvis, thin renal parenchyma, multilocular and dilated renal calyces with abundant gelatinous mucus, multiple stones measuring approximately 20 × 15 × 9 mm and three cauliflower-like masses measuring approximately 20 × 18 × 8 mm in the renal pelvis, and calyces (**Figure 2D**). Postoperative pathology suggested a high‐grade mucinous neoplasm of the renal pelvis and mucin residing partly in the interstitium of the cyst walls, without the involvement of the ureter, blood vessels, perirenal lymph nodes, or perirenal fat (**Figures 2E, F**). Immunohistochemistrical results showed that CDX2 (+), Villin (+), GATA3(−), P63 (−), CK7 (−) and Ki-67 (about 60%+), indicated features of appendiceal mucinous neoplasm metastasis to the renal pelvis (**Figures 3A–F**). The postoperative pathological stage was pT1N0M0. In the subsequent multidisciplinary consultation, salvage residual ureterectomy with a bladder cuff and chemotherapy were proposed, but both were refused by the patient. Perioperatively, no intestinal injury or any other complications occurred. The patient was satisfied with the treatment. The patient was discharged 7 days postoperatively and was closely followed up for 14 months. During the follow‐up period, no secondary infection, intestinal obstruction, metastasis, or recurrence was observed, while the serum levels of CEA and CA‐199 returned to normal.

## 3 Discussion

AMN is a rare type of gastrointestinal tumor with a low incidence, accounting for less than 1% of all cancers and 0.2%–0.3% of appendectomy specimens (2, 7). The incidence is similar between sexes, with a peak age of onset between 50 and 60 years, but the pathogenesis remains unclear. Mucinous cystic lesions of the appendix were first described by Rokitansky in 1842 as “mucoceles of the appendix” and were characterized as a swollen appendix filled with mucus (8). Malignant tumors of the appendix include neuroendocrine tumors, mucoepithelial tumors, lymphoma, goblet/pregoblet cell or complex carcinoid, adenocarcinoma, lymphoid or stromal sarcoma. In particular, neuroendocrine tumors and adenocarcinoma (mucinous, signet ring cell, or nonmucinous) represent approximately 65% and 20% of these malignant tumors of the appendix, respectively (2, 8, 9). Considering the rarity of these diseases and the lack of uniform clinical classification, the pathological characteristics and biological behavior of intraperitoneal dissemination are very difficult to describe. In 2016, the Peritoneal Surface Oncology Group International (PSOGI) classified appendiceal mucinous epithelial tumors of non‐neuroendocrine origin into five categories: i) LAMN, ii) high‐grade AMN (HAMN), iii) mucinous adenocarcinoma (MAC), iv) poorly differentiated signet ring cell adenocarcinoma (signet ring cells ≤ 50%), and v) signet ring cell carcinoma (signet ring cells > 50%). Among them, LAMN is the most common, accounting for approximately 60%–70% of all cases (10).

Achieving early diagnosis and accurate differentiation of the AMN pathological type is necessary for patient survival. LAMNs lack specific clinical manifestations; hence, most cases appear to be benign tumors at the early onset and need to be differentiated from other diseases, such as appendicitis, appendiceal perforation, and right lower abdominal masses. In advanced stages, LAMNs are manifested by intraperitoneal mucinous ascites or gastrointestinal adhesion-induced symptoms, such as chronic abdominal pain, abdominal distension, anemia, malnutrition, and intestinal obstruction (7, 11). Regardless of the specific classification, when the thin muscle layer of the appendiceal mucosa ruptures and mucin infiltrates through the appendiceal wall, AMNs can progress and induce peritoneal metastasis. An intraoperatively ruptured or residual mucinous tumor may be implanted in the peritoneum and eventually lead to peritoneal pseudomyxoma (PMP) (12, 13). Currently, surgery is the optimal treatment option for AMN (8), ensuring a complete abdominal exploration and tumor integrity preservation. Nevertheless, given the challenge of making an accurate preoperative diagnosis of a malignant AMN and identifying the typical characteristics of implantation metastasis, clinicians should be scrupulously careful when imaging findings suggest the presence of AMN or PMP. Moreover, considering that appendiceal tumors share the same origin as colorectal tumors, AMN may cooccur with colorectal cancer (CRC) (14). Therefore, preoperative colonoscopy is required to closely observe the appendiceal orifice, provide accurate pathology on biopsy, and rule out the possibility of concurrent CRC, facilitating the surgeons in selecting appropriate surgical strategies and determining the extent of surgical resection. Simple appendectomy can produce favorable clinical outcomes for LAMN without any extra‐appendiceal disease. In addition, changes in the survival rates of patients with positive surgical margins after extended colectomy have been demonstrated to be insignificant (15). AMN with local peritoneal metastasis is managed using the internationally accepted standard treatment regimen of cytoreductive surgery (CRS) with or without early postoperative intraperitoneal chemotherapy (EPIC) and hyperthermic intraperitoneal chemotherapy (HIPEC) to achieve longer survival (16, 17). In the present case, AMN was suspected when the CTU examination revealed appendiceal thickening, intraluminal hypodensities, and increased appendiceal wall thickness. Subsequently, colonoscopy and biopsy were ordered according to the treatment principles. Given that the pathology on biopsy met the diagnostic criteria for LAMN, standard appendectomy, abdominal exploration, and intraoperative freezing microtomy of resection margins were performed to ensure nontumorous tissue resection around the tumor. Moreover, with the postoperative pathology being consistent with the preoperative diagnosis, the treatment outcomes were favorable.

Most of the malignant tumors of the renal pelvis emerge from the transitional epithelium, as observed in approximately 90% of urothelial carcinomas, 10% of squamous cell carcinomas, and less than 1% of adenocarcinomas (18), among them, mucinous neoplasms of the renal pelvis are exceptionally rare, and was first reported by Ackerman in 1946 (19). However, its pathogenesis is still unclear, but it is generally believed that chronic infectious diseases, hydronephrosis, horseshoe kidney or kidney stones lead to obstruction and urothelial injury, which stimulate urothelial regeneration and repair, and then lead to glandular metaplasia of transitional epithelium, which gradually transforms into intestinal epithelium containing goblet cells, columnar cells and Pan’s cells. On this basis, the cells further dysplasia and development of cancer, resulting in adenocarcinoma or MAC (20–23). These tumors may exhibit symptoms similar to those of renal stone-induced pyonephrosis; such symptoms include lower back pain, low-grade fever, hematuria, and pyuria. Imaging findings typically show an enlargement of the affected kidney, with renal sinus and pelvis dilation, renal cortex thinning, and concomitant renal stones but without distinctive space‐occupying lesions. Given the lack of characteristic clinical manifestations, these conditions can be easily misdiagnosed as renal hydronephrosis or pyonephrosis with renal stones.

Primary mucinous neoplasms generally arise in the gastrointestinal tract or ovaries and are less likely to develop in the urinary system. Cases of AMNs with gynecological tumors of the ovaries, breasts, or endometrium have been reported (24). Unlike MAC of the appendix, LAMN is an indolent disease characterized by noninvasive growth and extremely limited distant metastasis (7, 25). Synchronous mucinous neoplasms of the renal pelvis and the appendix have not yet been reported. Hence, we cannot determine whether these neoplasms are both primary or if one has metastasized to the other. MAC of the renal pelvis may be diagnosed as primary only after ruling out the possibility of metastasis from the appendix, pancreas, colon, rectum, or ovary (26–29). Our patient first underwent appendectomy, and postoperative pathology suggested LAMN. The pathological findings and elevated serum levels of CEA and CA-199 indicated a strong possibility of AMN with secondary mucinous neoplasm of the renal pelvis. Despite all these important findings, the mechanism of LAMN metastasizing to the renal pelvis remains poorly understood. Further research is needed to confirm the potential association of metastasis with persistent obstruction and infection caused by renal stones.

Patients with primary renal pelvis MAC generally have a poor prognosis, with a survival rate of 2-5 years within the follow‐up period (30, 31). Similar to transitional cell carcinoma, this primary MAC is managed by radical nephroureterectomy with a bladder cuff (4, 5, 28). Intraoperative renal rupture-induced cancer cell dissemination and inadequate surgical resection usually lead to recurrence and metastasis. Our patient underwent preoperative nephrostomy, which led to the drainage of a substantial amount of gelatinous material. This substance was collected for exfoliative cytology, which confirmed the absence of malignant cells in triplicate sample analyses. However, the patient underwent laparoscopic right nephrectomy because she was misdiagnosed with calculi and pyonephrosis of the right kidney according to the indistinctive clinical symptoms, standard examination of the gelatinous material, and imaging findings. Postoperative pathology suggested that the patient had high-grade mucinous neoplasm of the right renal pelvis, with free surgical margins in the ureter. With the pathology report, residual ureterectomy with a bladder cuff and chemotherapy were recommended to minimize the risk of local recurrence and metastasis caused by residual tumor cells in the ureter. Unfortunately, the patient refused both options. Although the patient’s mucus exfoliative cytology was negative for 3 times before surgery, we still considered the possibility of malignant tumor cells in the mucus before surgery, so we performed more careful and removed the tumor as completely as possible during the operation. Considering the large volume of mucin in the opened specimen postoperatively, intraperitoneal chemotherapy using 0.4 g of 5‐fluorouracil and 500 ml of normal saline was administered for 2 h immediately after surgery to prevent peritoneal implantation metastasis caused by possible intraoperative mucin spillage. Thus, the physicians need to follow up with the patient and observe the benefits of EPIC in preventing PMP formation induced by the renal pelvis mucinous neoplasm and prolonging survival (32). Fortunately, during the 14-month close postoperative follow‐up, the patient showed no evidence of recurrence or metastasis, with the serum CEA and CA-199 levels returning to normal.

## 4 Conclusions

Currently, the accurate diagnosis of neoplastic lesions in the renal pelvis is still challenging, and undoubtedly needs to rely on pathological diagnosis. We need to rule out not only the common urothelial carcinoma in the pelvis, but also the rare renal cell carcinoma or metastatic tumor in the renal pelvis (33, 34). In this case, synchronous mucinous neoplasms of the renal pelvis and the appendix are indeed uncommon and have not yet been reported. Thus, developing an effective diagnosis and treatment plan is challenging. Limited by a lack of treatment experience, an insufficient understanding of mucinous neoplasms, and inadequate divergent thinking, renal pelvis mucinous neoplasm might be treated inappropriately. To ensure the right choice of surgical approach and achieve a favorable prognosis, clinicians need to confirm the origin and nature of the neoplasm through rapid freezing microtomy when they detect mucin in the renal lesion intraoperatively. Although it is important to careful resection of the neoplasm with absolute discretion during the operation and ensure the principle of tumor free, long-term close follow-up and monitoring after surgery is also an essential part of improving the prognosis.

## Data availability statement

The original contributions presented in the study are included in the article/supplementary material. Further inquiries can be directed to the corresponding author.

## Ethics statement

The studies involving human participants were reviewed andapproved by Ethics Committee of the First Affiliated Hospital ofGannan Medical College. The patients/participants provided theirwritten informed consent for the publication of this case report.

## Author contributions

YZ and XX prepared and wrote the article. YZ was directly involved in the management of the patients. QW and CZ were responsible for the collection and organization of the literature. QL revised the manuscript and acted as corresponding authors. All authors contributed to the article and approved the submitted version.
